# Advantages of genome sequencing by long-read sequencer using SMRT technology in medical area

**DOI:** 10.1007/s13577-017-0168-8

**Published:** 2017-03-31

**Authors:** Kazuma Nakano, Akino Shiroma, Makiko Shimoji, Hinako Tamotsu, Noriko Ashimine, Shun Ohki, Misuzu Shinzato, Maiko Minami, Tetsuhiro Nakanishi, Kuniko Teruya, Kazuhito Satou, Takashi Hirano

**Affiliations:** Okinawa Institute of Advanced Sciences, Uruma, Okinawa Japan

**Keywords:** PacBio RS II, Extra-long reads, De novo assembly, Targeted sequencing, Structural variations

## Abstract

PacBio RS II is the first commercialized third-generation DNA sequencer able to sequence a single molecule DNA in real-time without amplification. PacBio RS II’s sequencing technology is novel and unique, enabling the direct observation of DNA synthesis by DNA polymerase. PacBio RS II confers four major advantages compared to other sequencing technologies: long read lengths, high consensus accuracy, a low degree of bias, and simultaneous capability of epigenetic characterization. These advantages surmount the obstacle of sequencing genomic regions such as high/low G+C, tandem repeat, and interspersed repeat regions. Moreover, PacBio RS II is ideal for whole genome sequencing, targeted sequencing, complex population analysis, RNA sequencing, and epigenetics characterization. With PacBio RS II, we have sequenced and analyzed the genomes of many species, from viruses to humans. Herein, we summarize and review some of our key genome sequencing projects, including full-length viral sequencing, complete bacterial genome and almost-complete plant genome assemblies, and long amplicon sequencing of a disease-associated gene region. We believe that PacBio RS II is not only an effective tool for use in the basic biological sciences but also in the medical/clinical setting.

## Introduction

Genome sequencing technologies have extraordinarily progressed since the human genome project (HGP) was completed in 2003 [1, 2]. First-generation sequencing platforms (Sanger sequencers), used in the HGP, needed long run times, were expensive, and provided limited throughput (about tens of kbp per run) [3]. Second-generation sequencing (SGS) platforms, released in the mid-2000s, reduced run times and costs (around $1000 for the human genome) and increased throughput (about hundreds of Gbp per run) [2, 3]. SGS platforms have deepened understanding of genomes; however, regions such as high/low G+C regions, tandem repeat regions, and interspersed repeat regions are hard-to-sequence using SGS platforms, as well as Sanger sequencers, that produce short reads (35–1000 bases) and require PCR amplification [2, 3].

PacBio RS II (Pacific Biosciences, Menlo Park, CA, USA) is the first commercialized third-generation sequencer and was marketed in 2011. It utilizes a novel and unique single molecule real-time (SMRT) technology [3]. Thus, PacBio RS II is able to sequence single DNA molecules in real-time without means of amplification such as PCR, enabling direct observation of DNA synthesis by DNA polymerase. SMRT technology offers four major advantages compared to first- and second-generation platforms: (1) long read lengths (half of data in reads >20 kb and maximum read length >60 kb; our best record is 92.7 kb as of Nov. 2016), (2) high consensus accuracy (>99.999% at 30× in coverage depth, free of systematic errors), (3) low degree of bias (even coverage across G+C content), and (4) simultaneous epigenetic characterization (direct detection of DNA base modifications at one-base resolution). These advantages enable resolution and analysis of hard-to-sequence regions in complex genomes [4].

PacBio RS II is ideal for whole genome sequencing, targeted sequencing, complex population analysis, RNA sequencing, and epigenetics characterization. With this powerful platform, we have sequenced genomes of many species, from viruses to humans. Herein, we summarize and review our genome sequencing projects, including full-length sequencing of influenza A, complete genome assembly of bacteria such as multidrug-resistant tuberculosis, almost-complete genome assembly of the adzuki bean, and long amplicon sequencing of a gene associated with Stevens–Johnson syndrome. We believe that PacBio RS II is an effective tool not only for basic biology analyses but also critical in the medical/clinical setting.

## PacBio RS II’s real-time long read sequencing capacity

### Mechanism and performance

Single molecule real-time sequencing begins with preparation of SMRTbell template library. SMRTbell template is a double-stranded DNA template capped by hairpin adapters at both ends; hence, the template is structurally linear and topologically circular (described below). In SMRT sequencing, high-quality and sufficiently long DNA sample is expected so as to obtain good result. Typically, input DNA is sheared into 20 kb prior to the template library preparation and the library is size selected to remove shorter templates. The SMRTbell template library is then loaded into SMRT Cell, a 1-cm squared nanofabricated consumable chip comprising arrays of 150,000 wells on the surface, called zero-mode waveguides (ZMW) [5]. The size of each ZMW is 50 nm in diameter and 100 nm in depth. A single polymerase is fixed at the bottom of each ZMW and binds to the template [5, 7]. Then, four fluorescently labeled nucleotides are introduced into the ZMW chamber [5]. As each ZMW is illuminated from below, the wavelength of the light is too large to allow it to efficiently pass through the waveguide. Attenuated light (evanescent light) from the excitation beam penetrates the lower 20–30 nm of each ZMW [6]. This tiny detection volume provides 1000-fold improvement in the reduction of background noise [6]. After the polymerase incorporates a labeled nucleotide and cleaves its fluorophore, a light pulse corresponding to the incorporated base is produced in thin region [5, 7]. Each pulse has its own color intensity and duration time, and hence the type of the base is identified. This process occurs in parallel in up to thousands of ZMWs that make up the SMRT Cell. The maximum sequencing time for continuously collecting data from a SMRT Cell is limited by the life time of the specialized polymerase. It is 360 min at present and a template is sequenced at the speed of the polymerase in bases per second. Therefore, single molecule sequencing in real-time is enabled [5] and extra-long reads (maximum >60 kb and average 20 kb) are obtained without PCR amplification [5, 8]. By this SMRT technology, PacBio RS II can resolve hard-to-sequence regions such as AT/GC-rich regions and large structural variations, including insertions, deletions, inversions, translocations, duplications, and tandem/interspersed repeats [5, 8–10].

### Applications

#### De novo assembly

De novo assembly is a primary application of PacBio RS II [5]. The hierarchical genome assembly process (HGAP) for long reads generated by the PacBio RS II sequencer is developed to allow the complete and accurate shotgun assembly of bacterial-sized genomes [5, 11]. HGAP consists of three steps [11]. First, longer reads are preassembled by all other reads [11]. Second, the draft genome is constructed using highly accurate preassembled reads [11]. Lastly, the draft genome is polished by all reads [2, 11]. The error model of PacBio RS II’s data is random, and thus with sufficient depth of coverage, final consensus accuracy is in excess of 99.999% (QV of >50) [2].

#### Circular consensus sequencing (CCS) reads

As described above, the template for PacBio RS II sequencing is created by ligating hairpin adaptors to both ends of double-stranded DNA molecules, and thus it acts like a single-stranded closed circle. The polymerase used for sequencing has strand-displacement capacity. Therefore, for a short enough template, the polymerase can potentially circle around the template multiple times. [2, 5, 12, 13]. The consensus sequence of multiple paths yields a CCS read that is highly accurate (99.999%) as CCS effectively reduces random errors [5, 12–14]. The CCS is effective for full-length cDNA sequencing and targeted sequencing without assembly [5, 12].

#### Methylation characterization

DNA methylation can influence various processes such as gene expression, gene silencing, host–pathogen interactions, and transcriptional regulation [5]. Bisulfite sequencing is the most common sequencing method on SGS platforms for genome-wide detection of methylation patterns [5]. It needs to sequence both bisulfite-treated and untreated DNA, and needs to compare both sequence reads [5]. PacBio RS II, in contrast, does not require base conversion within the source material to detect base modifications. Instead, the kinetics of base addition is measured during the normal course of sequencing of an intact DNA. Thus, PacBio RS II can directly detect base modifications including some types of methylations (m6A, m4C, and m5C) by SMRT sequencing [5, 14].

## Noted studies using PacBio RS II

### Infectious diseases

#### Tuberculosis (*Mycobacterium tuberculosis* Kurono)

Tuberculosis (TB), caused by *Mycobacterium tuberculosis*, is one of the most prevalent and deadly bacterial infections affecting humans, with 8.6 million new cases and 1.4 million deaths annually worldwide [15, 16]. Moreover, the World Health Organization has estimated that 350,000 of these annual deaths are associated with HIV coinfection [15]. It is estimated that 310,000 patients newly diagnosed with pulmonary tuberculosis in 2011 were infected with multidrug-resistant (MDR) bacteria, with 9% of these patients having extensively drug-resistant (XDR) tuberculosis [15]. The highest TB burden regions are in Asia, with an estimated 58% of patients living in Asia in 2013 [16]. All Asian countries except for Japan are categorized as high or relatively high-burden countries, which is defined as more than 100 patients per 100,000 persons in the population [16]. To note, Japan’s national surveillance showed that the TB prevalence rate in Tokyo was 25 patients per 100,000 in population, which was more than twice the rate found in rural areas [16].

*Mycobacterium tuberculosis* (Zopf) Lehmann and Neumann (ATCC35812) (Kurono) is a strain isolated from human sputum in 1951 in Tokyo, Japan. Due to its consistently moderate virulence, it has been widely used as a standard virulent laboratory strain for virulence and immunization studies primarily in Japan. We used PacBio RS II to completely sequence the *M. tuberculosis* Kurono genome, and the single, circular contig (4,415,078 bp; G+C content of 65.60%) was determined [17]. The genome contained high G+C regions (2000 bp; G+C content of 80% maximum) and 117 sets of >1000 bp identical sequence pairs (unpublished). These findings provide the genomic foundation for future research using *M. tuberculosis* Kurono, especially in animal models.

#### Nosocomial infection of multidrug-resistant *Acinetobacter baumannii* IOMTU433

The emergence of multidrug-resistant (MDR) *Acinetobacter baumannii* has become a serious medical problem worldwide. We characterized the genetic and epidemiological properties of MDR *A. baumannii* isolated from a hospital in Nepal. In this study, 246 *Acinetobacter* spp. isolates were obtained from different patients. PacBio RS II completely sequenced the genome of one of these isolates (IOMTU433), which was obtained from a patient who had a history of acute exacerbation of chronic obstructive pulmonary disease with hypertension, type II diabetes mellitus, and hypothyroidism [18]. The genome belonged to a novel sequence type and clonal complex and harbored several drug-resistant genes. Two circular contigs representing a single chromosome (4,000,970 bp; G+C content of 39.15%) and a single plasmid (189,354 bp; G+C content of 39.53%) were obtained. The genome contained 41 sets of >1000 bp identical sequence pairs (5355-bp maximum) (unpublished). The MDR isolate harbored genes encoding carbapenemases (OXA and NDM-1) and a 16S rRNA methylase (ArmA). The use of PacBio also determined gene locations in the MDR isolate.

#### Nosocomial infection of multidrug-resistant *Pseudomonas aeruginosa* NCGM1984

A carbapenem-resistant *Pseudomonas aeruginosa* strain, NCGM1984, producing IMP-type metallo-β-lactamase (IMP-34) was isolated in 2012 from a hospitalized patient in Japan. PacBio RS II was used to sequence *P. aeruginosa* NCGM1984 [19]. One circular contig representing a single chromosome (6,850,954 bp; G+C content of 65.96%) was obtained. Complete genome sequencing revealed that NCGM1984 harbored two copies of blaIMP-34 located at different sites on the chromosome. The genome contained 6 sets of >10,000 bp identical pairs (27,239-bp maximum) (unpublished), which were prophages. Such regions are difficult to reconstruct by short-read sequencers, such as SGS platforms; however, PacBio RS II resolved the long identical pairs and will help elucidate the genome evolution by horizontal gene transfer.

#### Leptospirosis (*Leptospira interrogans* serovar Manilae strain UP-MMC-NIID)

*Leptospira interrogans* is a highly motile, obligate aerobic spirochete that causes leptospirosis in humans and animals, including wildlife, livestock, and pets. Leptospirosis is one of the most widespread (re)emerging zoonotic diseases in the world, particularly prevalent in tropical and subtropical regions. Humans and animals become infected through environmental, occupational, or recreational activities involving contact with infected urine or contaminated water or soil.

We used PacBio RS II to perform whole genome sequencing, de novo assembly, and DNA methylation detection of virulent and avirulent variants of *L. interrogans* to elucidate the pathogenomic mechanisms underlying leptospirosis [20]. We reported the complete genome sequences of low-passage virulent and high-passage avirulent variants of pathogenic *Leptospira interrogans* serovar Manilae strain UP-MMC-NIID [20]. A summary of the statistics for both variants is shown in Table 1.

**Table 1:** List of genomes sequenced on PacBio RS II on the Okinawa genome projects

Sample name	Methods	Replicon name	Genome length (b)	G+C content (%)	Hard-to-sequence regions	Accession no.	Published year [Ref.]
*Mycobacterium tuberculosis* Kurono (ATCC35812)	PacBio	Chromosome	4,415,078	65.60	G+C content of 80% region (2,000 bp), 117 sets of >1000-bp identical sequence pairs	AP014573	2015 [17]
Multidrug-resistant *Acinetobacter baumannii* IOMTU433	PacBio	Chromosome	4,000,970	39.15	41 sets of >1000-bp identical sequence pairs (5355-bp maximum)	AP014649	2015 [18]
		Plasmid	189,354	39.53		AP014650	
Multidrug-resistant *Pseudomonas aeruginosa* NCGM1984	PacBio	Chromosome	6,850,954	65.96	6 sets of >10,000-bp identical sequence pairs (27,239-bp maximum)	AP014646	2016 [19]
*Leptospira interrogans* serovar Manilae strain UP-MMC-NIID 1	PacBio	Chromosome 1	4,238,972	35.00	Plasmid, methylation	CP011931	2015 [20]
		Chromosome 2	358,378	34.91		CP011932	
		Plasmid pLIMLP1	70,055	34.54		CP011933	
*L. interrogans* serovar Manilae strain UP-MMC-NIID 67		Chromosome 1	4,238,922	35.00		CP011934	
		Chromosome 2	358,377	34.91		CP011935	
		Plasmid pLIMLP1	70,055	34.54		CP011936	
*Helicobacter pylori* Oki102	PacBio	Chromosome	1,633,212	38.81	8227-bp identical pair, methylation	CP006820	2014 [21]
*H. pylori* Oki112		Chromosome	1,637,925	38.81		CP006821	
*H. pylori* Oki128		Chromosome	1,553,826	38.97		CP006822	
*H. pylori* Oki154		Chromosome	1,599,700	38.80	G+C content of 28.7% region (2000 bp)	CP006823	
*H. pylori* Oki422		Chromosome	1,634,852	38.83		CP006824	
*H. pylori* Oki673		Chromosome	1,595,058	38.82		CP006825	
*H. pylori* Oki828		Chromosome	1,600,345	38.80		CP006826	
*H. pylori* Oki898		Chromosome	1,634,875	38.83		CP006827	
Influenza virus Okinawa strain	PacBio	cDNA	Data not published	Data not published	Full-length sequencing of all eight segments without assembly or resequencing	Data not published	This article
*Streptomyces versipellis* 4083-SVS6, *vst* gene cluster	PacBio	BAC	124,623	70.74	G+C content of 76.2% region (2000 bp)	LC006086	2014 [30]
*Actinomadura fulva* subsp. *indica* ATCC 53714, *flv* gene cluster	PacBio	Not opened	Not opened	Not opened	Not opened	LC095592	2015 [31]
*Lactobacillus curvatus* FBA2	PacBio	Chromosome	1,848,756	42.1	43 sets of >1000-bp identical sequence pairs (3118-bp maximum), G+C content of 26.9% region	CP016028	2016 [32]
*Dehalococcoides. mccartyi* IBARAKI	PacBio, SOLiD3	Chromosome	1,451,062	47.00	39 sets of >1000-bp identical sequence pairs	NZ-AP014563	2014 [33]
Endosymbiont of *Bathymodiolus septemdierum* str. Myojin Knoll	PacBio, 454, Sanger, Illumina	Chromosome	1,469,434	38.70	Subpopulation	AP013042	2015 [34]
Bacterial symbiont “TC1” of *Trimyema compressum*	PacBio	Chromosome	1,586,453	32.8	207 sets of >1000-bp identical sequence pairs, G+C content of 23.5% region	CP014606	2016 [36]
		Plasmid	35,795	29.7		CP014607	
*Vigna. angularis* cv. ‘Shumari’	PacBio, Illumina	11 Chromosome	All scaffolds Total 522,761,097 (cover 95%) Anchored scaffolds Total 471,245,712 (cover 85.6%)	–	Repetitive regions (50.6% of genome)	AP015034-AP017294	2015 [37]
*Salmonella enterica* subsp. *enterica* serovar Typhimurium strain ATCC 13311	PacBio	Chromosome	4,793,299	52.20	5420-bp identical sequence pair	CP009102	2014 [41]
		Plasmid	38,457	40.70		CP009103	
*Staphylococcus aureus* subsp. *aureus* Rosenbach 1884 (DSM 20231^T^)	PacBio	Chromosome	2,755,072	32.86	29 sets of >1000-bp identical sequence pairs (,063-bp maximum), tandem repeats (384 bp × 5 copies)	CP011526	2015 [42]
		Plasmid	27,490	30.69		CP011527	
*Pseudomonas aeruginosa* DSM 50071^T^	PacBio	Chromosome	6,317,050	66.52	5288-bp identical pair, 183 tandem repeats (246 bp × 20.7 copies)	CP012001	2015 [43]
*Clostridium sporogenes* DSM 795^T^	PacBio	Chromosome	4,142,990	27.98	86 sets of >1000-bp identical sequence pairs (4911-bp maximum), 380 tandem repeats (369 bp × 8.5 copies maximum), variable number tandem repeat	CP011663	2015 [44]
CM-SJS/TEN-associated *IKZF1* SNPs region of Japanese reference, Japanese SJS, and healthy subjects	PacBio	Targeted long amplicon	Data not published	Data not published	Targeted region (17 kb) of diploid genome	Data not published	This article

We found that while there were no major differences between the genome sequences, the levels of base modifications, such as methylation, were higher in the avirulent variant than the virulent variant. In addition, we discovered a novel plasmid in this species. Thus, PacBio RS II effectively assessed the methylation landscape and revealed a plasmid in the genome of this *Leptospira* species.

#### Gastrointestinal diseases (*Helicobacter pylori* Okinawa strains)

*Helicobacter pylori* is a spiral-shaped, Gram-negative, microaerophilic bacterium that colonizes the stomach. Approximately half of the world’s population harbors the bacterium. While *H. pylori* infection occurs worldwide, prevalence greatly varies among populations, and the vast majority of infected patients are asymptomatic. However, *H. pylori* infection is linked with the development of certain gastrointestinal diseases.

Although there is no significant difference in *H. pylori* prevalence between Okinawa, the southernmost prefecture of Japan (42% in 2004), and other areas in Japan, the incidence of gastric cancer in Okinawa is by far the lowest in Japan. We determined the genotypes of cagA and vacA virulence factors and revealed an association between these *H. pylori* virulence factors and gastroduodenal diseases in Okinawa. We performed whole genome sequencing and DNA methylation detection for eight *H. pylori* Okinawa strains, isolated from patients with gastrointestinal disease, using PacBio RS II to gain broader insight into *H. pylori* virulence [21]. A summary of the genome statistics is shown in Table 1. The oki102 genome contained 5 sets of >1000 bp identical sequence pairs (8227 bp maximum) (unpublished). The genome of oki154 contained low G+C regions (2000 bp; G+C content of 28.7% minimum) (unpublished). Furthermore, methylation analysis identified virulence factor-dependent motifs. We demonstrated that PacBio RS II is effective in determining the genomic profiles of critical virulence factors and methylation.

#### Full-length sequencing of influenza virus Okinawa strains

Influenza, commonly known as the flu, is a respiratory infectious disease caused by an influenza virus [22]. Seasonal influenza results in approximately 500,000 deaths worldwide per year [23]. In addition to annual seasonal epidemics, there have been four major flu pandemics: the 1918 Spanish flu, the 1958 Asian flu, the 1968 Hong Kong flu, and the 2009 flu pandemic [22]. The 1918 Spanish flu pandemic was the most catastrophic, causing more than 50 million deaths worldwide [23]. The 2009 flu pandemic approximately caused more than 200,000 deaths during the first 12 months of its circulation [22–25]. The first fatal case of the 2009 flu pandemic in Japan was identified in Okinawa [25, 26].

The three major genera of influenza virus, types A, B, and C, are all capable of infecting humans [23]. Types A and B are largely responsible for annual epidemics, whereas type C causes sporadic and mild infections [23]. Influenza A viruses are divided into various subtypes based on the viral surface glycoproteins hemagglutinin (HA) and neuraminidase (NA) [22]. HA and NA are the major antigenic proteins; there are 18 HA (H1–H18) and 11 NA (N1–N11) subtypes of influenza A viruses that can potentially form 144 HA and NA combinations [22]. Only a single subtype of influenza B virus has been identified [23].

The influenza A and B virus genomes are composed of eight single-stranded negative-sense RNA segments [23]. RNA viruses generally have very high mutation rates compared to DNA viruses, because viral RNA polymerases lack the proof-reading ability of DNA polymerases [27]. Influenza viruses have two major mechanisms of antigenic evolution, namely antigenic drift and antigenic shift. Antigenic drift occurs when the virus accumulates mutations at antigenic sites within HA or NA segments during error-prone replication. Antigenic shift occurs when a virus acquires an antigenically novel combination of HA and NA segments upon coinfection with two or more strains. This significant genetic diversity allows rapid adaptation to dynamic environments and evolved resistance to available vaccines and antiviral drugs [27]. Therefore, high-quality full-length genome sequences of influenza viruses are critical for developing successful vaccines and novel antiviral drugs. The lengths of viral RNA segments range from approximately 850–2350 bp [22, 24, 28]. PacBio RS II is able to determine each full-length sequence without any assembly or resequencing processes, because its read lengths are extremely longer than the segment lengths, while the data from SGS platforms need to be processed.

We performed full-length sequencing of 48 influenza viruses (37 strains of type A and 11 strains of type B) using PacBio RS II. These viruses were clinically isolated in Okinawa and collected by the Okinawa Prefectural Institute of Health and Environment between 2001 and 2013, including 2009 H1N1 pandemic viruses (AH1pdm09 Okinawa strains). cDNA was synthesized by multi-segment reverse transcription-PCR (M-RTPCR) from extracted RNA. Three of the type B strains were not amplified. All eight segments for the remaining strains (37 strains of type A and 8 strains of type B) were followed by 2 kb library construction using P4-C2 or P5-C3 chemistry without shearing. Two SMRT cells were sequenced per library with 120 min or 180 min movie time using PacBio RS II. CCS reads were generated using the Reads of Insert protocol in SMRT Analysis.

Full-length genome sequences for all eight RNA segments were obtained as the corresponding CCS reads with high consensus accuracy. Our genomic data set contained temporal and spatial information about the seasonal and pandemic prevalence of flu in Okinawa. Such insight gleaned will help elucidate the mechanism of acquired resistance to vaccines and drugs and thus inform future drug and vaccine development.

### Bioresources

#### Bacteria-derived drugs (*Streptomyces versipellis* 4083-SVS6 and *Actinomadura fulva* subsp. *indica* ATCC 53714)

Actinobacteria are well known for their ability to produce various bioactive natural products, with *Streptomyces* as prominent species [29]. High-quality genome assembly is necessary to obtain biosynthetic gene cluster sequences, which may glean insight into naturally derived products [29]. However, a potential obstacle in such assembly is the abundance of high G+C regions, which are difficult to determine by SGS [2].

Polyketide compounds constitute a large group of natural products that are important drug resources due to their highly diversified structures. Among them, versipelostatin (VST) is an unusual 17-membered macrocyclic polyketide product that contains a spirotetronate skeleton. The entire VST biosynthetic gene cluster (*vst*), spanning 108 kb, from *Streptomyces versipellis* 4083-SVS6 was identified by PacBio RS II, and the complete sequence of the cluster (124,623 bp; G+C content of 70.74%) was obtained [30, 31]. The genome contained high G+C regions (G+C content of 76.2%, 2000 bp) (unpublished). Via our accurate sequence data, we identified a novel cluster member, VstJ, that encodes for an enzyme that catalyzes [4 + 2]-cycloaddition. PacBio RS II can resolve the high G+C regions not elucidated by SGS platforms; thus, PacBio RS II may be useful in further drug discovery from natural products.

Additionally, the entire fluvirucin B_2_ biosynthetic gene cluster (*flv*) from *Actinomadura fulva* subsp. *indica* ATCC 53714 was identified by PacBio RS. Fluvirucins are 14-membered macrolactam polyketides with demonstrable antifungal and antivirus activities. The identified gene cluster contains three polyketide synthases, four characteristic β-amino acid-carrying enzymes, one decarboxylase, and one amidohydrolase. In our work, we clarified substrate specificity of the β-amino acid-selective adenylating enzyme FlvN.

#### Lactic acid bacteria (*Lactobacillus curvatus* FBA2)

*Lactobacillus* is the largest and most diverse genus among lactic acid bacteria (LAB). Their natural habitat ranges from fermented dairy, meat, and plant products to the oral cavity and intestinal and vaginal tracts of humans and animals. *Lactobacillus curvatus* is a LAB that is most commonly associated with fermented products. The *L. curvatus* strain FBA2 examined herein was isolated from radish and carrots pickled with rice bran and salt. FBA2 was selected from 200 LAB strains given its enhanced expression level of type I collagen and hyaluronan in human dermal fibroblasts. Use of FBA2 in skin-improving products was patented, and certain food products containing FBA2 are commercialized in Japan.

A single circular contig representing one chromosome (1,848,756 bp; GC content of 42.1%) was obtained using PacBio RS II [32]. No plasmids were detected by assembly or gel electrophoresis, indicating that FBA2 does not include plasmids. The genome contained 43 sets of >1000 bp identical sequence pairs (3118-bp maximum), including 22 insertion sequences and low G+C regions (2000 bp, G+C content of 26.9% minimum). Such regions are difficult to reconstruct by short-read sequencers as previously mentioned. Multi-kilobase reads in PacBio RS II resolved >1000-bp identical sequence pairs. The complete genome sequence of *L. curvatus* FBA2 will help to elucidate the skin-improving mechanism of and the diversity among *L. curvatus* strains.

#### Dechlorinating bacteria (*Dehalococcoides mccartyi* IBARAKI)

The chlorinated solvents, tetrachloroethene (PCE) and trichloroethene (TCE), are among the most abundant groundwater contaminants worldwide. *Dehalococcoides* reduces chlorinated solvents by producing ethane from the solvents.

A *Dehalococcoides*-containing bacterial consortium that was able to dechlorinate cis-1, 2-dichloroethene to ethene was obtained from the sediment mud. To obtain detailed information of the consortium, the metagenome was analyzed using an SGS platform, SOLiD 3, and PacBio RS II. We determined the full-length circular genome sequence (1,451,062 bp; G+C content of 46.99%) of *Dehalococcoides* sp. in the consortium, and named it *D. mccartyi* IBARAKI [33]. Its genome contained 39 sets of >1000-bp identical sequence pairs (unpublished). We highlighted that the combination of SGS and PacBio RS II yielded a detailed metagenomic profile that may better inform bioremediation efforts to abolish chlorinated solvents from groundwater.

#### *Bathymodiolus septemdierum* Myojin knoll vent mussel endosymbionts

The vent mussel *Bathymodiolus septemdierum* was collected from deep-sea hydrothermal vents in the Izu-Ogasawara area of Japan using Hyper Dolphin, a remotely operated vehicle. The vent mussel lives in symbiosis with chemoautotrophic bacteria in gill bacteriocytes and uses the bacteria as inorganic carbon sources. The metabolic ability of the symbionts is largely affected by the geochemical conditions, whereas the physicochemical features of the hydrothermal vent environment are highly variable, thus availability of metabolic substrates is unpredictable. Therefore, genomic information is needed to elucidate the metabolic landscape of these symbionts.

We performed whole genome sequencing of vent mussel endosymbionts using 454 and Sanger sequencing platforms and re-sequenced using PacBio RS II and Illumina platforms [34]. The complete sequence of the cluster (1,469,434 bp; G+C content of 38.7%) was obtained. We found that a single symbiotic bacterial species had several heterogeneous subpopulations in host bacteriocytes. Long reads by PacBio RS II were able to capture this heterogeneity unlike the other platforms, such as the short reads of Illumina. Particularly, our PacBio data showed that the symbiont population was composed of at least four subpopulations possessing one of the heterogeneous genomes, each of which had or lacked gene clusters for key metabolic enzymes such as hydrogenase and nitrate reductase. The genomic heterogeneity among the subpopulations may enable differential utilization of diverse substrates. Such findings advance our understanding of metabolic acclimation and genomic evolution in symbiotic bacteria. PacBio RS II will be useful to detect subpopulation of viruses and bacteria.

#### Ciliate symbiont (bacterial symbiont “TC1” of *Trimyema compressum*)

Trimyema ciliate (*Trimyema compressum*) lives in anaerobic fresh water environment and harbors methanogenic archaea and a bacterial symbiont named “TC1” in its cytoplasm. Metabolic interactions among the organisms are still unknown. Genomic information of those organisms will reveal the interactions. However, in the previous study using 454 sequencer and Illumina, TC1 was reconstructed as 106 contigs, and the maximum length of the contigs is about 110 kb [35]. Therefore, we carried out new sequencing using PacBio RS II. As the result, two circular contigs representing a single chromosome (1,586,453 bp, G+C content of 32.8%) and a single plasmid (35,795 bp, G+C content of 29.7%) were obtained [36]. The genome contained 207 sets of >1000-bp identical sequence pairs and low G+C regions (2000 bp, G+C content of 23.5% minimum) (unpublished). Such regions are difficult to reconstruct by short-read sequencers. The result will accelerate the understanding of the symbiotic relation.

### Plants

#### Azuki bean (*Vigna angularis* cv. “Shumari”)

Azuki bean (*Vigna angularis*) is the second-most important grain legume in East Asia [37]. In these times, the breeding of azuki bean is widely conducted and is targeting seed quality, cold tolerance, and disease resistance. Although this species was recently sequenced (estimated genome size 540 Mb), the draft assembly covered ~70% of the genome. Here, we sequenced the azuki bean (*V. angularis* cv. “Shumari”) genome using PacBio RS II, in addition to Illumina [37].

We obtained a near complete genome (11 pseudomolecules, total size 471,245,712 bp, and covering 85.6%, in anchored scaffolds) and achieved the best contiguity and coverage among currently assembled legume crops. The PacBio assembly produced 100 times longer contigs with 100 times smaller amount of gaps compared to the SGS assemblies. The genome contained repetitive regions of 273 Mb (50.6%). Such regions are difficult to reconstruct by short-read sequencers. These data will greatly support breeding activities including cloning and maker-assisted selection in azuki bean. PacBio RS II can resolve repetitive regions, as shown in this study.

### Microbial genomic reference materials

#### Overview

The US Food and Drug Administration (FDA) highlighted the need for reference materials (RM) and methods that would permit performance assessment in approving the assay for clinical use of the SGS [38, 39]. As the result, the FDA collaborated with the National Institute for Standards and Technology (NIST) to develop RM consisting of whole human genome DNA [38–40]. NIST is developing reference material, which is HapMap/1000 Genomes CEU female NA12878 (RM8398), with the Genome in a Bottle Consortium [38–40]. Also, NIST is developing four microbial genomic RM for microbial sequencing; *Salmonella enterica* LT2, *Staphylococcus aureus*, *Pseudomonas aeruginosa*, *Clostridium sporogenes*. As the previous study, we sequenced four bacteria by PacBio RS II, which are type strains of the same species as the microbial genomic RM. The four complete genomes were obtained using PacBio RS II.

#### *Salmonella enterica* subsp. *enterica* serovar Typhimurium ATCC 13311

Salmonella, the leading bacterial food-borne pathogen, encompasses a large group divided into 6 subspecies and more than 2500 serovars. *S.* Typhimurium causes human gastroenteritis and mouse typhoid. Its multidrug-resistant strains have spread worldwide. *Salmonella enterica* subsp. *enterica* serovar Typhimurium strain ATCC 13311, a quinolone-susceptible strain, has been used as a reference in multidrug resistance studies.

We reported the first complete sequence of *S.* Typhimurium ATCC 13311 obtained using PacBio RS II [41]. Two circular contigs were obtained, one representing a chromosome (4,793,299 bp, G+C content of 52.2%) and the other, a plasmid (38,457 bp, G+C content of 40.7%). The genome contained 5420-bp identical sequence pairs. This complete genomic sequence of *S.* Typhimurium ATCC 13311 will accelerate investigations into multidrug resistance.

#### *Staphylococcus aureus* subsp. *aureus* DSM 20231^T^

*Staphylococcus aureus* is a Gram-positive, non-spore-forming, and nonmotile coccus. It is a human indigenous bacterium, and about 30% of healthy humans harbor it in their nasal passages. *S. aureus* is the major cause of staphylococcal disease. The emergence of antibiotic-resistant forms of pathogenic *S. aureus* is a worldwide problem in clinical medicine.

The type strain of *S. aureus* subsp. *aureus* Rosenbach 1884 (DSM 20231^T^) was first isolated in 1884 from human pleural fluid by Rosenbach. We determined the first complete genome sequences using PacBio RS II [42]. Two circular contigs were obtained, one representing a chromosome (2,755,072 bp; average G C content, 32.86%; maximum, 60%), and the other representing a plasmid (27,490 bp; GC content, 30.69%). The genome contained 29 sets of >1000-bp identical sequence pairs (3063-bp maximum) and several notable tandem repeats (e.g., 384 bp × 5 copies and 18 bp × 42 copies). The complete genome sequences of the type strain of *S. aureus* subsp. *aureus* reported here can be used as the standard reference for the species and will accelerate the understanding of the pathogenomic characteristics of the species and their role in (antibiotic-resistant) staphylococcal disease.

#### *Pseudomonas aeruginosa* DSM 50071^T^

*Pseudomonas aeruginosa* is an aerobic, motile, and Gram-negative rod-shaped bacterium that exists in a wide range of ecological niches. It is a major opportunistic human pathogen and is also an important causative agent of hospital-acquired nosocomial infections, characteristically in immunocompromised individuals. The emergence of antibiotic-resistant forms of *P. aeruginosa* is a worldwide problem in clinical medicine.

We reported the first complete genome sequence of *P. aeruginosa* DSM 50071^T^ determined by PacBio RS II [43]. A single circular contig representing a chromosome was obtained (6,317,050 bp, average G+C content of 66.52%, and 843_coverage). The genome contained ten sets of >1000-bp identical pairs (5288 bp maximum) and 183 tandem repeats (246 bp × 20.7 copies maximum).

The complete genome sequence of the *P. aeruginosa* type strain reported here can be used as the standard reference for the species and will accelerate the understanding of the pathogenomic characteristics of the species, especially in (antibiotic-resistant) *Pseudomonas* infection.

#### *Clostridium sporogenes* DSM 795^T^

*Clostridium sporogenes* is an anaerobic spore-forming bacterium that causes food spoilage. *C. sporogenes* is widely used as a nontoxigenic surrogate for *Clostridium botulinum* in the validation of food sterilization because of its physiological and phylogenetic similarity to *C. botulinum* and nontoxigenicity.

A single circular contig representing a chromosome was obtained (4,142,990 bp; average GC content, 27.98%) using PacBio RS II [44]. Recently, a sequence of *C. sporogenes* NCIMB 10696^T^, which originated from the same strain (McClung 2004^T^) as *C. sporogenes* DSM 795^T^, has been determined using 454, Illumina, and Sanger technologies (CP009225) (4,141,984 bp; average GC content, 28.00%). The genome contained 86 sets of >1000-bp identical sequence pairs (4911-bp maximum) and 380 tandem repeats (369 bp × 8.5 copies maximum). We found three marked differences between the sequences of DSM 795^T^ and NCIMB 10696^T^. First, in a 39-bp tandem region, DSM 795^T^ carried 25.5 copies, whereas 10696^T^ carried 20.5 copies. Second, in a 312-bp tandem region, DSM 795^T^ carried 5.9 copies, whereas 10696^T^ carried 4.9 copies. Third, DSM 795^T^ had a 501-bp extra region that could be inserted in 10696^T^. On DSM 795^T^ sequencing, the PacBio RS II platform produced extra-long reads with an average of 3959 bp and a maximum of 35,904 bp, and large numbers of reads completely covered those regions: 290 reads for the first, 191 reads for the second, and 359 reads for the third. This result suggests that the number of tandem repeats is underestimated in the 10696^T^ sequence.

PacBio RS II provides power for assessing structural variations such as variable number tandem repeat.

### Targeted long amplicon sequencing of CM-SJS/TEN-associated *IKZF1* SNPs region

**Fig. 1:**
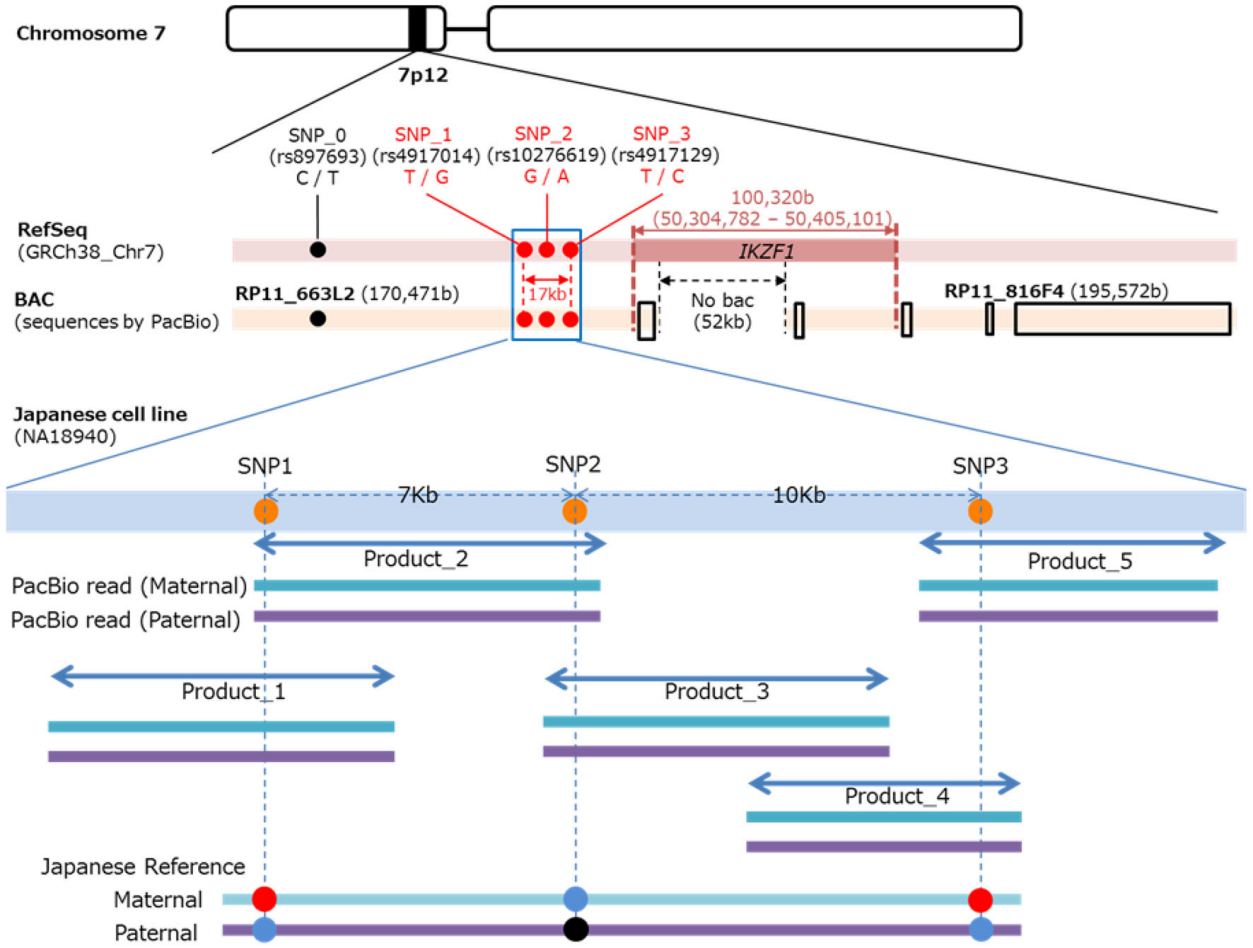
Targeted sequencing of CM-SJS/TEN-associated *IKZF1* SNPs region in Japanese using PacBio RS II. (1) Bacterial artificial chromosome (BAC) (RP11_663L2) sequences, including CM-SJS/TEN-associated *IKZF1* 3SNPs, were obtained by PacBio RS II sequencing. (2) Targeted region (17 kb) of Refseq (GRCh38_Chr7) sequence was validated by BAC (RP11_663L2) sequences. (3) RefSeq and BAC sequences had no differences in the targeted region (17 kb), including 3 SNPs. (4) Primers were designed based on RefSeq that cover target SNPs, where the expected size of each product was 8 kb. (5) DNA from Japanese (NA18940) cell line was amplified by PCR with the primer pairs. (6) Minimum number of primer pairs (5 products) were selected. (7) Long reads were produced by the PacBio RS II sequencing platform for the selected primer pairs. (8) PacBio RS II single molecule sequencing technology produced maternal and paternal reads separately for an individual. (9) Reference diploid genome sequence was then constructed with the maternal/paternal-molecule-originated reads

Stevens–Johnson syndrome (SJS) and its severe variant, toxic epidermal necrolysis (TEN), are extremely serious inflammatory vesiculobullous reactions of the skin and mucous membranes, including the ocular surface and oral cavity [45]. These reactions are often associated with inciting drugs, infectious agents, and cold medicines [45]. Although they are rare, with an annual incidence of 1–6 cases per million persons, these reactions carry high mortality rates of 3% for SJS and 27% for TEN, and surviving patients often suffer severe sequelae, such as vision loss [45]. HLA genotypes are associated with SJS/TEN [45]. As susceptibility gene for cold medicine-related SJS/TEN (CM-SJS/TEN) with severe mucosal involvement, including severe ocular complications, it was indicated that *IKZF1* single nucleotide polymorphisms (SNPs) were significantly associated in Japanese, Korea, and India subjects [45, 46]. *IKZF1* encodes Ikaros, which is a member of DNA-binding protein family and transcription factor and plays an important role in the development of several lymphocytes [45, 47]. *IKZF1* SNPs [rs4917014 (G vs. T), rs4917129 (C vs. T), rs10276619 (G vs. A)] play some role in the efficiency of *IKZF1* alternative splicing [45]. These 3 SNPs are located within 17 kb in the 5´region of *IKZF1* and the distance between the SNPs and *IKZF1* exon 1 is more than 50 kb (Fig. 1) [45]. The distance between rs491704 and rs10276619 is 7 kb and the distance between rs10276619 and rs4917129 is 10 kb (Fig. 1).

We performed targeted long amplicon sequencing of CM-SJS/TEN-associated *IKZF1* SNPs region (17 kb) using PacBio RS II for constructing haplotype-dependent reference sequence that is to be utilized to discover novel SNPs and screen them (Fig. 1). PacBio’s PCR-free, extra-long reads with high consensus accuracy provide highly accurate, allele-resolved long amplicon sequencing. First, a bacterial artificial chromosome (BAC) containing the three SNPs (RP11_663L2, 170 kb) was sequenced and assembled to validate the sequence of the targeted region in RefSeq sequence (GRCh38_Chr7). RefSeq and BAC sequences had no differences on the targeted region (17 kb). Second, five sets of primer pairs were designed based on the RefSeq sequence so that at least two amplicons would cover each SNPs, where expected size of the amplicons is 8 kb. Third, DNAs from Japanese (NA18940) and Caucasian (NA12878) cell lines from HapMap samples were amplified with the primer pairs. All of the products for each cell line showed good amplification. Lastly, the amplicons were converted into 10-kb library for P5-C3 chemistry without shearing. One SMRT cell per library was sequenced using PacBio RS II with 180-min movie time. CCS reads were generated using Reads of Insert protocol in SMRT Analysis. High-accuracy CCS reads were successfully clustered into two allele types, namely paternal and maternal allele types. CCS reads for each type were merged into single sequence and thus reference diploid genome sequences for Japanese population were generated. We sequenced DNAs from both Japanese SJS and healthy subjects and observed allele patterns of the three *IKZF1* SNPs for each group. Our method will definitely help improve a wide range of GWAS studies.

## Conclusions

In the human genome, approximately 50% of the entire sequence is comprised of a broad class of repetitive elements [48]. Although some repeats appear to be non-functional, others have played an important role in human evolution. Instability of repetitive sequences within the genome is associated with a number of human diseases. The repeats may consist of just two copies or millions of copies, and they can range in size from 1 to 2 bases to millions of bases. SGS platforms, which have become essential tools in genetic and genomic analyses, produce vast amount of data. However, the reads are too short to be identified within the data. PacBio RS II provides aforementioned advantages such as long read lengths, high consensus accuracy, low degree of bias, and epigenetic characterization, and this sequencing platform is able to resolve sequences with high/low G+C, tandem repeat, and interspersed repeat regions.

We have sequenced a large number of genomes using PacBio RS II and published many scientific publications with collaborators in Okinawa genome projects in a wide variety of area including medical area. For viral and prokaryotic genomes, we determined all eight RNA segments of influenza A virus as full-length sequences (data not published), 21 complete bacterial genomic sequences, and 2 entire bacterial biosynthetic gene clusters [17–21, 30–34, 36, 41–44]. For eukaryotic genomes, we constructed almost-complete (totally 95% of 540 Mb in 11 chromosomes) genome sequences of azuki bean [37]. Furthermore, we determined a highly accurate Japanese reference diploid genome sequence that led to the novel discovery of SJS/TEN-associated SNPs in the Japanese population (data not published).

PacBio RS II is a viable option for small, medium, and large genomes; however, there is still room for improvement. In a recent comprehensive assembly of the gorilla genome using PacBio RS II, the large, complex, gene-rich structural variant events spanning hundreds of kilobase pairs were detected [9]. Although the majority of full-length common repeats were resolved, heterochromatin and large segmental duplications remained mostly unresolved given that read lengths were not sufficiently long enough to cover these repetitive structures [9]. Irys (BioNano Genomics, San Diego, CA, USA) is a next-generation physical genome mapping system that provides long-range information of genome structures. Irys directly measures structural variants and repeats within long, single molecule “reads” for comprehensive analysis. Thus, the combination of genome sequencing by PacBio RS II and genome mapping by Irys may allow for high-quality hybrid assembly of complex genomes [10]. PacBio RS II has significantly impacted basic science and biology and is reaching its influence into the clinical/medical atmosphere. PacBio RS II along with Irys will accelerate research towards precision medicine via its combined holistic and comprehensive view of genomes, transcriptomes, and epigenomes.
